# The meaning of dignity when the patients' bodies are falling apart

**DOI:** 10.1002/nop2.301

**Published:** 2019-05-22

**Authors:** Vibeke Bruun Lorentsen, Dagfinn Nåden, Berit Sæteren

**Affiliations:** ^1^ Department of Nursing and Health Promotion OsloMet – Oslo Metropolitan University Oslo Norway

**Keywords:** body, dignity, life experience, nursing, palliative care, patient

## Abstract

**Background:**

People with advanced cancer disease experience great bodily changes due to disease or treatment. They tend to feel ashamed when their bodies are subjected to such changes and they feel their dignity is threatened.

**Aim:**

To explore the patients' experiences of the bodily changes in relation to dignity.

**Design:**

The study has a hermeneutic qualitative design.

**Method:**

Individual in‐depth interviews and participant observations were conducted with 13 patients with advanced cancer disease at a hospice inpatient unit in Norway. Gadamer's ontological hermeneutics inspired the interpretation.

**Results and conclusion:**

The patients' unpredictable, sick bodies forced the patients, or gave them the opportunity, to relate to their bodies in an honest way. The patients, living in interaction between suffering and health, strove to find dignity. The patients had a will to live and they experienced a love in their unruly bodies that both helped alleviate their suffering and give them an experience of enhanced dignity. It is important that nurses have insight into the consequences of bodily changes for the patients' experiences of dignity in health and suffering to provide good, dignified care.

## 1 INTRODUCTION

Serious illness leads to a loss of “the destination and map” that previously guided the sick person's life (Frank, 2013). The “map” that changes is, among other things, the body. People with advanced cancer diagnoses experience great bodily changes due to disease and/or treatment. The changes are, among others, emaciation, scars, hair loss, fatigue, ulcers, loss of limb(s), but also invisible changes in bowel, urinary and sexual function (Gobel, Yarbro, & Wujcik, 2011; Price, 2009).

The most frequently reported symptoms occurring in patients with cancer in a palliative phase are fatigue, feeling drowsy, difficulty sleeping and worrying (Stark, Tofthagen, Visovsky, & McMillan, 2012). Pain (Oechsle, Goerth, Bokemeyer, & Mehnert, 2013) and weight loss (Teunissen et al., 2007) are also reported as frequent symptoms.

Research studies also explore how patients experience living with advanced stage cancer and the existential challenges concerning living with the disease at the end of life (García‐Rueda, Carvajal Valcárcel, Saracíbar‐Razquin, & Arantzamendi Solabarrieta, 2016; Henoch, Danielson, Strang, Browall, & Melin‐Johansson, 2013; Sæteren, Lindström, & Nåden, 2011, 2015). Research studies describe a perception of the body as a stranger and as an alien. The body sets boundaries and limits for existence and does not feel like a home anymore, but rather like a prison where battles are fought (Lindwall & Bergbom, 2009; Sekse, Gjengedal, & Råheim, 2013). Even though the bodily changes may seem fundamentally threatening to the human being, few studies have delved deeply into how people with advanced cancer disease experience living in a changed body.

### 1.1 Background

The concept of bodily dignity is rarely addressed in research, except dignity as related to identity (Jacobson, 2007; Nordenfelt, 2004). The body is described as the bearer of relative dignity (Edlund, Lindwall, Post, & Lindström, 2013) and people may experience dignity when they perform actions that are in accordance with their culture's and their body's rules and norms for dignity. Embodied dignity is described as when a person affirms the value of his or her carnal nature and a certain comportment, showing both a self‐accepted vulnerability and a certain honour in this, is noticeable (Galvin & Todres, 2015).

When the body is sick and weak, bodily dignity cannot be related to the performance of actions that are in accordance with a healthy and strong body. People have to relate to their bodies in a different way: as something they are, rather than something they have (Merleau‐Ponty, 2002), when their bodies are more or less falling apart.

The concept of dignity is important in nursing because it says something profound about the status, the sacredness and the uniqueness of the human being (Lorentsen, Nåden, & Sæteren, 2016; Tranvåg, Synnes, & McSherry, 2016). Research studies reveal that dignity is threatened and people feel ashamed when they experience great bodily changes caused by their severe cancer disease (Franklin, Ternestedt, & Nordenfelt, 2006; Lin, Watson, & Tsai, 2013). The body becomes unrecognizable, it smells from wounds that will not heal and the patients experience a lack of control of bodily functions. These bodily changes affect not just the patients' bodies, but their whole life.

Tranvåg and McSherry (2016) claim that nurses have an intuitive understanding of dignity. However, they may lack an in‐depth understanding of the underlying components of the concept, an understanding which would have better enabled them to identify and resolve practices that violate dignity. Since the literature review reveals that the body influences dignity (Edlund et al., 2013; Galvin & Todres, 2015), knowledge about the patients' experiences of their bodily changes would probably yield knowledge of some of these underlying components of dignity. Through the patients' stories, we hoped to gain insight into the affective knowledge that requires both mind and body when living with advanced cancer and into how this provides deeper knowledge of the phenomenon of dignity from an inside, bodily perspective. Merleau‐Ponty's philosophy of the body, as the basis for consciousness and language (Merleau‐Ponty, 1968, 2002), is also important in our effort to understand more about the subject of the study.

## 2 AIM

The primary aim of this study is therefore to explore the patients' experiences of bodily changes in relation to dignity.

The secondary aims are as follows:

- What are the patients' experiences of the bodily changes?
- How do the patients' experiences of bodily changes give insight into the phenomenon of dignity?

## 3 METHODOLOGY

### 3.1 Design

Since the aim of the study is to acquire a deeper understanding of the patients' experiences of the bodily changes in relation to dignity, the study has a hermeneutic qualitative design based on Gadamer's (2004) ontological hermeneutics, where the purpose is to achieve understanding through interpretation. Gadamer (2004) does not offer a special method of interpretation, but has described how new understanding emerges through slow, lingering reading of a text, where pre‐understanding and data merge into a fusion of horizon.

### 3.2 Setting, sample and method of data collection

Data were collected through individual in‐depth interviews and participant observations. Thirteen patients, four men and nine women, aged 53–83, from a hospice inpatient unit in Norway, participated in the study. The patients included had an advanced cancer diagnosis, were at the end of life and experienced bodily changes due to disease and/or treatment (Table 1). All patients spoke and understood Norwegian and were mentally aware of time and location. The period of data collection lasted from January–June 2017. One of the head nurses selected the participants in accordance with the inclusion criteria.

**Table 1:** Age and gender of the patients

Patient	Gender	Age
1	Woman	83
2	Woman	57
3	Woman	60
4	Woman	53
5	Man	60
6	Woman	75
7	Man	77
8	Man	79
9	Woman	56
10	Woman	75
11	Man	70
12	Woman	57
13	Woman	55

### 3.3 Qualitative interviews

The first author (V.B.L) conducted the interviews with the patients in the patient room at the hospice inpatient unit. An interview guide was applied (Table 2) and the interviews started with an open‐ended question, asking the patients to tell about their bodily experiences of the illness. Other research questions focused on how the body had changed during the illness trajectory and the meaning of those bodily changes for the patient and for relations with other people. The interviews lasted between 35–70 min. Data from the interviews were taped and transcribed by the first author. The research group consisted of three Norwegian researchers who, from previous studies, were familiar with both the subject of the research and the research method.

**Table 2:** The interview guide

I am concerned about the body. As a result of illness and/or treatment, many patients with advanced cancer disease experience bodily changes *Can you tell me about the bodily experiences you have had in relation to being sick?* *‐ Have you experienced that your body has changed as a result of illness and/or treatment?* *Can you say something about how your body has changed throughout the course of the disease?* *What do the bodily changes mean to you?* *Do the bodily changes affect how you see yourself?* *Do the bodily changes have any importance for your relationship with others?* *Do you have any thoughts on how we, as nurses, can help you/the patients to have good bodily experiences?*

### 3.4 Participant observation

The first author (V.B.L) observed different care situations at the hospice inpatient unit: patients interacting with nurses or doctors, receiving treatment from the physiotherapists, or conversing with the occupational therapists. Since our primary duty as researchers is to treat the patients with respect and caution, we found it ethically proper to follow and observe each patient during a 1‐day shift only, while most of the patients had a 14‐day stay. For the same reason, the observer did not stay permanently in the patient's room during the whole day shift but went in and out and came in when various health personnel visited the patient. Thus, each patient was observed for a total of approximately 3–4 hr.

The primary purpose of the observations was to give the researchers more background knowledge about the suffering body to gain a deeper and better understanding of the patients' situation. This helped the first author, in the interview situation, gain a deeper insight into what was at stake for the patients. Also, that the researcher spent more time with the patients probably made the patients more familiar with the researcher and more confident and open in the interview situations.

We did not develop an observation guide as we did not want to focus on specific situations but absorb as much as possible in the situations (Nåden, 2010). The first author observed how the patients talked about the illness, their use of body language, the tone of voice, the way they moved their bodies, slow or fast, the cooperation with other health personnel and what was explicitly put into words or left implicit.

### 3.5 Interpretation

Even though Gadamer (2004) did not outline a specific method of interpretation, he offered some key concepts, which have guided our interpretation. These are openness to the text, questions and answers, the hermeneutic circle, parts and whole and fusion of horizons. The texts were read several times to grasp the overall meaning. To search for a deeper meaning and to achieve a movement from the empirical to a generic understanding, three questions were posed to the texts: “What does the text say?”, “What does the text mean?” and “What is the deeper meaning in the text?” The questions were organized in a matrix. Throughout the repeated readings of the text, there was a dynamic and inner movement between the steps in the matrix or between the parts of the text and the whole. The researchers' pre‐understanding was challenged, and the process continued until consensus was reached in the research group.

### 3.6 Ethical considerations

The Norwegian Social Science Data Services, reference number 42503, approved the project. A head nurse in the hospice inpatient unit informed the patients about the purpose of the study. She obtained written consent from the participants before the research study started and informed the participants about the principle of voluntariness of participation, the duty of confidentiality, anonymity and the possibility of withdrawal from participation without giving any reason.

The research raised special ethical issues as the patients were weak and at a vulnerable stage in their life. Thus, the ethical principle to do no harm was important throughout the research project. The first writer was particularly concerned with this during the interviews, but also during the participant observations and strove to be sensitive and to remain aware of the patients' conditions. Thus, the researcher asked the patients frequently if they needed breaks, or wanted to finish the interviews, or if they wanted the researcher to leave the room. The researcher also made sure that the staff would follow‐up the patients afterwards if the interviews evoked feelings or reactions that they needed to talk about. However, spending time with the participants throughout the participant observations not only entailed a risk of causing harm; it also could potentially strengthen the patients' confidence and thus have a beneficial effect.

## 4 RESULTS

The patients told stories about how their bodies changed due to the advanced cancer disease and what the bodily changes meant to them and their surroundings. Telling their stories helped the patients get in touch with their own vulnerability; they could not trust their bodies in the same way as before. The unpredictable bodies threatened the patients' identity and dignity and they became afraid of losing themselves. However, the bodily changes also revealed a strong desire to live and to feel that their bodies still functioned. Four themes emerged from the hermeneutic interpretation:

- The unruly body – a wreckage that rocks the depth of being.
- The body as conveyor of existential truth.
- The life‐affirming will discovered in the wrecked body.
- Moving into new and health‐inducing rooms in their sick bodies.

### 4.1 The unruly body – a wreckage that rocks the depth of being

The patients had to relate to bodies that were no longer a protection or something they could rely on, but rather caused painful feelings and even fear:

There is nothing I can do with my body. It is a mystery. Things happen that are outside of my control because I am being eaten up inside. My body is like a horror movie. It looks normal, but there is a lot of bubbling and boiling inside of it. (2)

Several participants cried when we asked them how they experienced their altered body. A man described his body like wreck, shit and a bunch of stuff that was impossible to repair.

The voiced fear of annihilation while still alive also emphasized what was really at stake for the patients. A woman described her fear of whether her dignity would disappear someday, while she gradually felt she lost herself and the more disgusting parts of death would take over.

The impossibility of stopping the life‐changing conditions that the illness caused reinforced the threat to dignity, while predictability and control have elements of protection that all human beings long for. A man said:

After the last operation, new tumours pop up faster than the doctors are able to get rid of the former. They lie near the surface and spring up like small mushrooms. (5)

The uncontrollable body also had consequences for how the patients enjoyed social life. They used a lot of strength to try to control a body that was impossible to handle.

However, despite these stories, several participants confirmed that they were not worried that changes in their appearance would be a threat to their identities:

In the beginning, I felt sad when I looked at myself in the mirror. I don't feel that anymore. I have seen my body too much for that. It is as it is. The important thing for me now is that my body carries me. (10)

Nevertheless, when observing some of the female patients, the contrasts in their appearance struck us and suggested to us that maybe appearance still had some importance for dignity. A woman had beautiful fingers with red nail polish and earrings that gleamed. She had no hair; her skin was pale and her face was quite oedematous without any make‐up. She told us how thoroughly she tried to keep her looks and fix what was possible to fix. Another woman said:

I try to keep my dignity as long as possible. I comb my hair and try to keep my looks and take care of myself… which many probably forget because they think there is no point. It is of great importance to me to take care of myself. (6)

### 4.2 The body as conveyor of existential truth

The altered body was no longer able to cover up or ignore problems the way it did when it was healthy. The sick body was rather a guide that showed and let the patients understand, what was at stake, a conveyor of truth.

A woman described the truth that the body represented as a new stability in her life that made her more honest with herself. Others said that their dependency on others forced them to relate to their surroundings and to themselves in a closer and more honest way. They had to relate to their body or vulnerability to a greater extent than earlier because the previous shelter no longer worked. A woman said:

I think I have become more humble because I feel I am going to die quite soon. I have also become more dependent on others because I am so weak and miserable. I am probably more honest than before. (12)

The bodily changes were also described as an existential journey. One woman described it as discovering new layers of truth in herself. The patients also said that the bodily changes had forced them to reduce their activities and to understand and give priority to what was important in their lives:

The problems with my legs help me realize that I cannot do everything anymore, but the most important things I can still do. If I didn't have any pain in my legs, I would probably have gone along and never stopped and I would have been too tired afterwards… The bodily changes have helped me calm down and become aware of what is important in life. (13)

However, the body was not only a conveyor of the good but also of the painful truth. Several participants said they had to move out of their house or apartment, or that they were not able to visit their cottage anymore because of the bodily changes:

I live on the third floor. I have great problems getting up the stairs to my apartment. So I have to move, probably to the opposite side of the district, because there they have some flats that will suit me. (7)

Being forced to move at the end of life might feel quite dramatic and laborious. The home was, for many participants, not just a house, but also a place of belonging, of memories and feelings that made it difficult to leave.

### 4.3 The life‐affirming will discovered in the wrecked body

Despite the body being at risk of collapsing, the patients showed a great desire, or a life‐affirming will, to keep their body constantly moving, keeping it in an upright position to ensure that it still lived and functioned:

I try to walk because my legs are beginning to fail. I feel that I have to try to keep the strength I have in my legs by walking. (10)

We also observed the importance of movement in patients who exercised, by squeezing small balls several times a day, despite that they had not experienced any effect yet. The patients showed a resoluteness and persistency when they exercised with the physiotherapists. They seemed to move into another mental state and used all their strength and concentration to accomplish the training.

To be active and involved when the prognosis was severe seemed to be important for the patients' experiences of dignity. One woman confirmed this and described the illness as a tango and said she was the man in the dance, leading the tango and the illness was not.

However, the patients also described it as tough to persistently work and uphold a body that just wanted to kneel and rest. Sometimes it was good just to be left alone with no expectations as to what to do.

### 4.4 Moving into new and health‐inducing rooms in their sick bodies

The patients also told stories about how they tried to take care of their unruly bodies, not only in a physical way but also in a psychosocial or mental way as well. One woman described how she tried to bring light into her life every day. The light was not anything in particular, but a kind of feeling of something good from the past. Another woman mentioned rituals as an example of light and claimed that the regularity, the firmness, but also the goodness of the rituals helped her to remain rooted when the body crumbled away.

The patients were concerned with exploring and moving into new health‐inducing rooms in their bodies as ways to endure their difficult life situation and preserve dignity:

I have started to think that my body is my house and that I still am situated in my body. I try to imagine nice pictures of my body. A house is a good place where you are happy, safe and taken care of. It is a place filled with warmth and love…. I have started painting. I feel that is important for my identity now… By doing this I feel that I have moved into new rooms deeper in my body. (3)

Imagination and painting helped the patient keep the life‐affirming force that love represented in her life, which was important for dignity and health. The will and the way the patients opened up for new and health‐inducing rooms in their bodies showed that they were responsible and willing to take care of themselves and make the best of a difficult situation to preserve dignity.

## 5 DISCUSSION

The initial interpretation of the results disclosed how the patients experienced their wrecked bodies, what the bodies represented and how the patients felt, related and acted to keep themselves and their bodies whole to preserve dignity. The results were further interpreted through a continuing dialogue with the researchers' pre‐understanding, the philosophy of the body, the theory of dignity and other research studies. This resulted in three overarching themes that gave a deeper insight into the phenomenon of dignity.

Themes 1–2 from the results showed how the patients, through their sick and wrecked bodies, were shaken to the depth of their being and thus gained the possibility of being in contact with the existential truth, which gave them insight into dignity from a bodily perspective. Thus, themes 1–2 were merged and the new theme that arose was:

- The unruly body – a possible source of a deeper understanding of dignity.
- The life‐affirming will discovered in the wrecked body showed a natural force to achieve health and dignity and the theme that emerged from the interpretation was:
- The life‐affirming will – a natural force for achieving health and experiencing dignity.

The patients' movement into new and health‐inducing rooms in their sick bodies showed how they rediscovered love, deeply rooted in themselves, as a healing power that alleviated suffering and promoted dignity. The theme that arose was:

- Love, as a healing power in the health‐inducing rooms, alleviating suffering and promoting dignity.

These themes will be discussed further.

### 5.1 The unruly body – a possible source of a deeper understanding of dignity

The sick bodies forced the patients, or at least gave them a possibility, to deal with their bodies in an honest and natural way. It was difficult to cover up or hide the bodily changes from themselves or from their surroundings, as the results reveal. The sick body and the world were in a continuous movement and interaction with each other and this seemed to make the patients relate to their bodies in a deeper and more truthful way.

Sæteren, Lindström, and Nåden (2011) claim that the human being is composed of extreme opposites like finite and infinite, mortal and immortal, freedom and necessity. The authors refer to Kierkegaard (1964), who says that the ethical demand is to become oneself by growing together with oneself. A process of striving for being and becoming a self (Eriksson, 2007) might take place in the interaction or movement between these apparently opposite life experiences that the patients described in our study. The patients' stories about a new stability and honesty in their life might be an example of such a process of becoming.

Understanding dignity through the lens of ambiguity or interaction between suffering and health means that dignity is not a concept of objective explanations, but rather a concept understood in the continuous movement and interaction between the opposite life experiences. The meaning of trying to understand dignity is therefore not to search for well‐defined categories, but rather to reflect on what the good ambiguity means to the human being. Bengtsson (2001) refers to Merleau‐Ponty (1968), who describes the good ambiguity as the ambiguity that is in continuous interaction and which is not abolished. Human beings are in this good interaction when they touch the world and further reconcile or acknowledge the uncertainty and possibilities that life gives (Gustafsson, Wiklund‐Gustin, & Lindström, 2011), the way the patients in our study did. Gadamer (1996) states that we must recognize and accept the fundamental disposition of anxiety in the face of life and equally of death and he describes this as ontological dignity.

### 5.2 The life‐affirming will – a natural force for achieving health and experiencing dignity

The patients described a great sensibility to the way their bodies functioned. They told several stories about how important it was to them that their bodies still felt alive and worked despite the descriptions of their bodies as wreckage. Active and vital bodies were important for their experiences of health and dignity and they were willing to push their sick bodies more than they otherwise would have done. This is confirmed by Wickström‐Grotell (2016) and Wikström‐Grotell and Eriksson (2012), who describe bodily movement as an absolute value related to human dignity and as a sign of life. Bodily movement gives a sense of being present and alive and induces trust and hope for the future.

The patients defied both pain and fatigue to feel and confirm that their bodies were alive. The natural will to feel alive was like an inner force (Gadamer, 1996; Nyholm, 2015), which kept the patients going.

This will becomes more visible in borderline situations when the human being senses life's finitude (Nyholm, 2015). However, this will may also be weakened by suffering, as suffering may prevent or make it difficult for people to listen to their inner will. The patients told quite detailed about their changed bodies. This might imply that they were in contact with and familiar with their bodily changes. The body was not a stranger to them anymore. This may be understood as that the patients had reached the level of becoming in Eriksson's ontological health model (Eriksson, 2006), implying that they were reconciled with their difficult life situations.

Thus, the life‐affirming will discovered in bodily movement was a positive force for achieving health and experiencing dignity. When the patients lived in harmony with their inner will, they were no longer operated by external circumstances, but were true to themselves.

### 5.3 Love as a healing power in the health‐inducing rooms, alleviating suffering and promoting dignity

The patients also described their strong will to discover new, health‐inducing rooms in their sick bodies as a way to take care of themselves and preserve their dignity. They told several stories about how they filled their life with goodness and love. Love was a healing power that alleviated suffering and could turn life from destruction to strength and hope. The patients experienced love as fundamental to being. They rediscovered the love deeply rooted in themselves and this helped them endure suffering. The love‐affirming will gave the patients strength to live. Thorkildsen, Eriksson, and Råholm (2013) confirm that love is above and beyond suffering and can turn life from destruction and coldness to strength and hope. Further, that no human being can exist without love (Thorkildsen et al.., 2013). Love is a precondition for dignity and a burning force that makes the good happen in each individual (Kaldestad, 2018).

It might seem as a paradox that the patients' awareness of their own death made them so aware of how to fill their life with goodness and love. However, other research studies confirm that when patients confront their own death, they awaken existentially. They strive for a wholeness of body, soul and spirit and for becoming a self, which entail experiencing integrity and dignity in life despite bodily changes (Arman & Rehnsfeldt, 2006; Sæteren et al., 2011; Sæteren, Lindström, & Nåden, 2015). Suffering develops the human being's ontological awareness (Thorkildsen et al., 2013).

### 5.4 Methodological considerations

The sample consisted of 13 patients from a hospice inpatient unit. Most participants were women. The results may have been different if more men had participated in the study.

Using observations in the collection of data may provide a richer understanding of the field than using only interviews, even if the observations are short. However, as described earlier, it was not ethically defensible to perform longer observations, as this might cause harm. The short observations helped the researcher develop trust among the patients and formulate better questions in the interviews, as the observations gave deeper insight into the patients' experiences of their vulnerable bodies.

The researchers have experiences and knowledge about the subject of the study, the empirical field, the research method and the study's theoretical perspective from previous work as researchers and teachers in nursing education. Thus, the researchers' pre‐understanding and the theoretical foundation of the study have consequences for the whole research process, both as to which results were explored and how the results were interpreted. Therefore, it has been important to ensure that the pre‐understanding does not cover up, or blinds us to, what emerges throughout the research process (Alvesson & Sköldberg, 2018). We have tried to keep our minds in a persistent uneasiness by constantly asking ourselves what we do not understand. This has helped us to keep our minds open and not conclude too early.

We also found it reasonable to use Gadamer's hermeneutic philosophy to understand the text, even though Merleau‐Ponty, who is important for understanding the body, represents a phenomenological tradition. This is because we use Gadamer and Merleau‐Ponty on different levels, the former is related to method and the latter is part of the theoretical foundation of the study.

## 6 CONCLUSION AND RECOMMENDATIONS

The patients' experiences of their bodily changes gave insight into the phenomenon of dignity. The sick bodies force the patients, or give them the possibility, to deal with their bodies in an honest way when they can no longer cover up or hide the bodily changes, not from themselves, nor from the surroundings. The patients live in an interaction between health and suffering and reconcile, or acknowledge, the possibilities that life gives. This yields insight into dignity as the good ambiguity.

Secondly, the life‐affirming will is discovered as a natural force in the wrecked body, a force that helps the patients achieve health and experience dignity. The patients willingly push their bodies and defy both pain and fatigue to feel and show that they are still alive and that their bodies are still able to carry them. Lastly, love is discovered as a healing power in the new and health‐inducing rooms that the patients explore in their sick bodies. A love that alleviates suffering and promotes dignity.

Exploring the concept of dignity through the patients' stories about their sick bodies might develop knowledge of dignity from an inner perspective, a knowledge which may comprise some of the intuitive understanding nurses have of dignity and which needs to be verbalized. Since dignity often is threatened when patients are seriously ill, it is important that the nurses do not abolish the interaction between health and suffering, but encourage and strengthen the patients to live in and relate honestly to, both their suffering and health to become themselves and experience dignity. Likewise, it is essential that the nurses help the patients to find their inner resources to manifest themselves and promote dignity, such as the “will for life” and “love as a healing power” discovered in this study.

Dignity is essential in nursing care and more attention needs to be paid to the concept and its articulation in clinical nursing practice and among nursing leadership to provide good, dignified nursing care.

Further research is also required to explore the relationship between the body and the phenomenon of dignity, including the nurses' perspective, as this little attention is paid to this in nursing research today.

## CONFLICTS OF INTEREST

The authors have declared no conflicts of interest.
